# Evaluating the Portable X-ray Fluorescence Reliability for Metal(loid)s Detection and Soil Contamination Status

**DOI:** 10.21203/rs.3.rs-3414584/v1

**Published:** 2023-10-12

**Authors:** Zain Alabdain Alqattan, Janick F. Artiola, Dan Walls, Mónica D. Ramírez-Andreotta

**Affiliations:** 1 Department of Environmental Science, College of Agriculture and Life Sciences, University of Arizona, Tucson, AZ, USA; 2 Department of Science and Technology Studies, Rensselaer Polytechnic Institute, Troy, NY, USA; 3 Division of Community, Environment & Policy, Mel and Enid Zuckerman College of Public Health, University of Arizona, Tucson, AZ, USA

**Keywords:** Metal(loid)s, Portable X-ray Fluorescence Spectroscopy (pXRF), Inductively Coupled Plasma Mass Spectrometry (ICP-MS), Pollution load index, Enrichment factor, Geo-accumulation index, Soil monitoring

## Abstract

Environmental Justice (EJ) communities may experience barriers that can prevent soil monitoring efforts and knowledge transfer. To address this gap, this study compared two analytical methods: portable X-ray Fluorescence Spectroscopy (pXRF, less time and costs) and Inductively Coupled Plasma Mass Spectrometry (ICP-MS, “gold standard”). Surface soil samples were collected from yards and gardens in three counties in Arizona, USA (N=124) and public areas in Troy, New York, USA (N=33). Statistical calculations, i.e., two-sample t-tests, Bland-Altman plots, and a two-way ANOVA indicated no significant difference for As, Ba, Ca, Cu, Mn, Pb, and Zn concentrations except for Ba in the two-sample t-test. Iron, Ni, Cr, and K were statistically different for Arizona soils and V, Ni, Fe and Al concentrations were statistically different for New York soils. To assess the degree of contamination, a pollution load index (PLI), enrichment factors (EF), and geo-accumulation index ($I_{\text{geo}}$) were calculated for both methods using U.S. Geological Survey soils data. The PLI were >1, indicating pollution across the two states. Between pXRF and ICP-MS, the $I_{\text{geo}}$ and EF in Arizona had similar degree of soil contamination for most elements except Zn in garden and Pb in yard, respectively. In New York, the $I_{\text{geo}}$ of As, Cu, and Zn differed by an order of magnitude between the two methods. The results of this study demonstrate that pXRF is a reliable method for the inexpensive and rapid analysis of As, Ba, Ca, Cu, Mn, Pb, and Zn. Thus, EJ communities may use pXRF to screen large numbers of soil samples for several environmentally relevant contaminants to protect environmental public health.

## 1. Introduction

Urbanization and industrial activities have increased the amounts of released contaminants and potential exposure routes for communities. These contaminants can accumulate in soil and impact human and ecological health. Mining for example, provides society with needed elements, but also serves as a primary source of heavy metal (HM) pollution (Fashola et al., 2016; Zhuang et al., 2009). In mineral-rich areas the soil environment may be naturally rich in or become a repository of inorganic contaminants diffused and emitted from nearby mining activities (Tepanosyan, et al., 2018). Exposure to heavy metals and metalloids can cause chronic health problems at low concentrations (Wragg, 2013; Haidar et al., 2023), such as learning disabilities, kidney dysfunction, endocrine disruption, and damage the nervous system (Gorini et al., 2014; Paschoalini et al., 2019). Arsenic, Cr and Ni are categorized as carcinogens by the International Agency for Research on Cancer, and Pb as a probable carcinogen (Kim et al., 2015; U.S.EPA IRIS, 2021, U.S.EPA IRIS, 2022); exposure to any concentration of Pb is unsafe (World Health Organization, 2019). Heavy metal exposure threatens humans, animals, and the ecosystems; HM are taken up by crops, ingested, and can bioaccumulate over time in organisms (Gitet et al., 2016), leading to behavioral disruption, infertility problems, and in severe cases, death (Hejna et al., 2018). In addition, metal(loid)s alter soil microbial communities and reduce vegetative coverage in terrestrial ecosystems by causing morphological abnormalities in plants (Tiwari & Lata, 2018; Amari et al., 2017) and limiting the microbial metabolism (Wang et al., 2020). Rural and urban communities have initiated agri-food systems like organic farming to maintain a sustainable food source (Measham, 2010) and use regional resources such as soil, land, and water. Therefore, the concerns about mining activities impacting food safety have increased because HM accumulation jeopardizes rural soils and the well-being of local and indigenous communities living nearby mining sites (Haddaway et al., 2019; Gibson & Klinck, 2005). To protect ecosystem and human health, an affordable monitoring technique is needed to detect metal(loid) concentrations in areas impacted by industrial and resource extraction waste sites.

**Figure 1.**
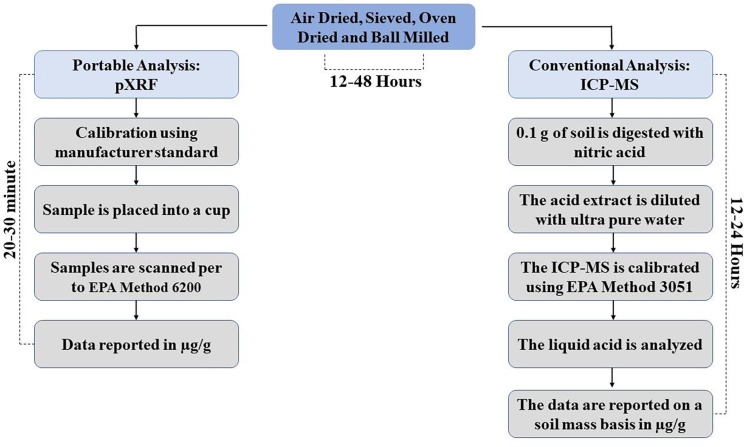
Soil Sample preparation and analysis comparison between pXRF and conventional analyses of ICP-MS. Note with ICP-MS, laboratory wait time and data report back to end-user will add additional time.

Due to cost, time, and access, currently, “gold standard” metal(oid)s soil methods of analysis are generally not available (Marguí, et al., 2013), especially to those who need it most. These methodologies include a lengthy acid digestion process and analysis via flame atomic absorption spectroscopy (FAAS), inductively coupled plasma emission spectrometry (ICP-OES), and inductively coupled plasma mass spectrometry (Figure 1). A low-cost alternative is the portable X-ray fluorescence (pXRF), which has proven to be a multi-elemental technique that can be applied in-situ with minimally processed samples to delineate heavily contaminated zones (Weindorf et al., 2013). Although the pXRF offers a rapid, lower-cost tool to screen soil and sediments for metal(loid)s; how does it compare to the laboratory “gold standards? As a case in point, the Center for Disease Control’s Agency for Toxic Substances (ATSDR) recommends using a pXRF for Soil Screening, Health, Outreach, and Partnership (soilSHOP) events designed to provide community members with free soil screenings (ATSDR, n.d.), but currently only recommends using the pXRF for lead. This is a missed opportunity to identify other possible soil contaminants and protect community health.

This study aims to assess whether the pXRF can serve as a reliable instrument to accurately determine lead, arsenic and other heavy metal concentrations in residential soils. Soils metal(loid) concentrations measured by ICP-MS and pXRF were statistically compared to determine the pXRF reliability in environmental assessments and provide an alternative detection method from the costly chemical analysis. Therefore, the insights gained from this comparison will provide a deeper understanding of the pXRF’s performance and reliability to serve as a tool for local communities to improve human and soil health.

## 2. Methods

### 2.1 Study and site description

This study is part of the University of Arizona Gardenroots project (https://gardenroots.arizona.edu/), which assesses residential environmental quality of communities neighboring resource extraction activities through a co-created citizen/community science design (Ramírez-Andreotta et al., 2013a, 2013b, 2015; Sandhaus et al., 2019; Manjón et al., 2020; Zeider et al., 2023). The research focuses on three counties in Arizona, USA, which are Apache, Cochise, and Greenlee, and the city of Troy in New York, USA.

Over 90 local community members were trained on how to properly collect garden and yard soil samples and 124 soil samples were submitted by Arizona community members. Arizona has nine abandoned hazardous or uncontrolled Superfund sites recognized as National Priorities List (NPL) by the U.S.EPA (Arizona Department of Environmental Quality, ADEQ, 2022). Apache and Greenlee do not have any superfund sites; however, they are home to 12 active mines (Richardson et al., 2019). The largest copper mining operation in North America is the Morenci mine in Greenlee County. The surrounding area is known to have high concentration of As, Cr, Cu, Pb, Mn, and Ni (U.S. Department of the Interior, 2020). The Apache, Cochise, and Greenlee counties are rural communities and have a population of 65,623, 126,050, and 9,404, respectively (U.S. Census Bureau, 2019). The percentage of individual older than 65 in Apache (16.9%,), Cochise (23.8%), and Greenlee (12.9%) and the poverty per person in Apache and Cochise is higher than the national poverty rate in the USA at 28.4% and 17.1%, respectively (Census Bureau, 2022a; 2019; U.S. Census Bureau, 2022b; 2019; U.S. Census Bureau, 2022c). Apache has an annual precipitation of 10.55 inches and a mean annual temperature of 52 °F; Cochise receives 14 inches of precipitation per year with an annual average temperature of 63.1 °F; Greenlee has a mean annual rain of 16 inches and an average annual temperature of 59 °F (NOAA National Centers for Environmental information, 2022).

Thirty-three soil samples were collected from Troy, New York. Troy is considered an urban city with a population of 50,760 (Census Bureau, 2021). The city has an annual rain and a mean temperature of 41 inches and 47 °F, respectively. Although there is no mining project nearby the city, still, many superfund sites were recognized by the U.S.EPA in Albany County such C&F Plating Company, Inc., which highlights the predicament of having potential released contaminants such copper in the region (U.S.EPA, n.d.c). In this context, Troy has high potential exposure to lead paint coming from housing units built before 1960; medium to high potential chemical accident management plan in some part of the city; high hazardous waste proximity which account to hazardous waste facilities in a 5 km radius (U.S.EPA, 2015).

### 2.2 Soil collection and field sampling

The Gardenroots participants were trained in sample collection protocols from their gardens and yards; the first is described as an area used to grow edible and ornamental plants, whereas yard is considered as native and unamended land where children’s practice physical activity and outdoor play. The participants picked six sampling spots arranged as a grid pattern in the garden area, close to growing spots of vegetables and other edible plants. The topsoil layer (6 inches) was loosened, homogenized, and then placed in a labeled 2-gallon bucket. The samples were mixed thoroughly in the bucket and separated into two labeled brown papers with the participant number and date of collection, then placed in a plastic bag (Zip bag). The participants chose the spots where they often play or walk for the yard samples. For yard soils, the same procedure was applied, using a different bucket. All samples were stored in a refrigerator immediately after collection, then transferred with dry ice into an insulated foam kit to process for expedited shipping. The same procedure was followed to collect soil samples from Troy, New York.

### 2.3 Soil pH and texture analysis

All soil samples from Troy, New York were analyzed for particle size and pH. A fisher XL-20 meter was used to measure pH value after calibration with three buffer values of 4,7, and 10. The procedure starts by adding 10 grams of dried soil from each sample into the vial that has 20 millimeters of 18 Mega ohm water. The vials were placed in the shaker for 30 minutes. The electrode prob was placed into the stirring samples (approximately 2 cm deep) to measure the pH. Throughout the process, the prob was rinsed and recalibrated after every 5 measurements. To determine sand, silt and clay size fractions in soils, the hydrometer method and triangle of textural classification were applied as per the USDA soil classification system (USDA, 1999).

### 2.4 ICP-MS Soil Analysis

All samples were air-dried for 24–96 hours, sieved to 2 mm diameter, then oven-dried for a constant mass at 105°C (VWR, gravity convection oven), ball milled to 80–100 μm (SPEX SamplePrep, 8000D), and stored in paper envelopes until analyzed. Each sample went through a microwave acid digestion process using the modified method of U.S. EPA Method 3051; 1 ml of concentrated nitric acid (Omni race HNO3, EMD Chemicals) was reacted with 0.1 g of the sieved soil for one hour at room temperature, then 1 mL of ultrapure water (18 MOhm) was added. The samples were sealed to run at high pressure and temperature via microwave digestion (CEM Model MARS6 microwave, Matthews, North Carolina). Each batch had a National Institute of Standards and Technology (NIST SRM 2711a Montana II soil) control sample. ICP-MS quantifiable detection limits for each element are provided in Table 1. Arizona soil samples were analyzed for Be, Na, Mg, Al, K, Ca, Ti, Mn, Cu, Co, Zn, As, Pb, Cr, Se, Mo, Ag, Cd, Sn; and New York soil samples for As, Ni, V, Cu, Cr, Al, Fe, Zn, Pb, and Mn. Moreover, concentrations below the detection limit were considered equal to half the method detection limit in reporting the soil elemental content.

### 2.5 pXRF Soil Elemental Analysis

The pXRF instrument (DELTA Premium Handheld XRF) used in this study was purchased from OLYMPUS, USA, and consisted of a 40kV tube and large-area silicon drift detector used mainly for detecting low levels of trace elements in soil and mining (Olympus Corporation, n.d.). The pXRF instrument is also equipped with optimized beam settings of 4W x-ray tube and 200 μA current, a rechargeable Li-ion battery, and automatic barometric pressure correction.

Prior to soil analysis, the internal X-ray stability was monitored per the guided manual by calibrating the lowest energy electron shell (Fe K-α) of 316 stainless steel coins before each run, which helps measure the count of the elements based on their oxide weight proportion. For quality assurance and control prior to usage, quality control and assurance, a SiO_2_ blank and NIST standard measurements were taken prior to sample analyses. Table 1 shows the manufacture’s LODs in part per million or microgram per kilogram (ppm, μg g^−1^) in the operational setting “geochemistry” and was used for calibrating the pXRF. DELTA PC Software configured the calibration modeling and beam operation to enhance data analysis. The general procedure followed the U.S.EPA Method 6200 intrusive analysis (U.S.EPA, 2007), and the Center for Disease Control/Agency for Toxic Substances and Disease Registry’s (ATSDR) soilSHOP protocol (ATSDR, 2022).

As done for ICP-MS analysis, Arizona’s soil samples were sieved, dried, and balled milled then analyzed via pXRF; whereas Troy’s Soil samples were only sieved and dried for the analysis. All the soil samples were analyzed in the laboratory by trained technicians using the pXRF, Gardenroots samples were screened for 19 elements, whereas Troy samples were screened for only 10 elements. All soil samples were individually stored in 6.5 × 5.9-inch Ziplock bags. Each sample was screened for 90 seconds at 3 discrete locations, ensuring the soil in the Ziplock bag is at least 1-inch thick at each screening point. If there was a high variation >20% between the three values, additional screenings were conducted to ensure the accurate measurement for each soil sample. Lastly, the average of the three screening results was calculated and recorded with the corresponding sample number in the logbook.

### 2.6 Data Analysis Methods

To validate the pXRF methodology, a series of statistical analyses were conducted between pXRF and ICP-MS measurements for each element of concern in this study, and the unit expressed in (μg g^−1^). The following ICP-MS below detection limits elements Mo, Co, Se, Ag, Sn, and Sb were excluded from the analysis. All statistical procedures used in this study were conducted via R-studio version 4.1.1, Adobe Photoshop version 22.4.2 and Microsoft excel 2016. Using mean concentration of each metal(loid)s, a two-sample t-test was performed first to test the null hypothesis that the average concentration of each metal(loid) concentration was the same for both methods. If the probability values were not significantly different (p > 0.05), then there is no variation effect observed between the two method’s elemental concentration.

Next, an intraclass correlation (ICC) was also performed as another approach to quantify the similarity between the two methods. A high ICC coefficient (close to 1) suggests high similarity between methods whereas a low ICC value (close to 0) indicates elemental concentrations were different depending on the method utilized, thus measuring the linear relationship between two continuous variables, where each concentration is scaled by mean and standard deviation.

To further compare the two methods and where one technique is considered the “gold standard”, in this case, ICP-MS, a Bland-Altman analysis was conducted to assess how similar the pXRF is to the ICP-MS. The x-axis represents the mean of each element for both methods and the y-axis represent the difference between the sampling method concentrations (Giavarina, 2015). Each plot has the average concentration represented as a horizontal line. The upper and lower lines represent the limits of agreement, meaning that if the differences are normally distributed, 95% of the data should be between these limits.

Based on the findings of the two-sample t-test and interclass correlation coefficients, a post-hoc testing using Tukey’s HSD for two-factor ANOVA was applied to the Arizona’s soil to further understand the variability of the pXRF data. It was hypothesized that soil amendment (unamended, yard and amended, garden) would contribute to the disparity in elemental concentration. To determine whether soil texture influenced pXRF performance, a Canonical Correlation Analysis (CCA) was conducted (Hardoon et al., 2004). The analysis describes the association between two data matrices which are soil texture and elemental concentrations by measuring the linear relationship while preserving the main facets of the correlation.

### 2.7 Enrichment, Accumulation, and Pollution Comparisons Methods

#### 2.7.1 Enrichment Factor

To evaluate the degree of pollution and whether the pXRF could reliably indicate enrichment, the pXRF and ICP-MS soil data was also used to calculate the enrichment factor. EF describes the presence of an element relative to the reference metric (Bern et al., 2019). The EF was calculated as:

(1) $$EF=\frac{\left[\frac{c_{n,\mathrm{sample}}}{C_{\mathrm{ref,sample}\;}}\right]}{\left[\frac{C_{n,\mathrm{background}\;}}{C_{\mathrm{ref,background}}}\right]}$$

where $C_{n}$ is the detected metal(loid) mean concentration by pXRF or ICP-MS in units of mg kg^−1^. The Mn mean concentration detected by the ICP-MS was set as the reference value $\left(C_{ref}\right)$, except for Mn calculation which has Fe mean concentration as the $C_{ref}$. All the background concentrations ($C_{n}$ and $C_{ref}$) were implemented based on element concentrations in soils determined by United States Geological Survey (Table 2, Shacklette and Boerngen, 1984). EF less than one indicate no enrichment; 1 < EF < 3 means a minor enrichment; 3 < EF < 5 describes a moderate enrichment; 5 < EF < 10 explains a moderately severe enrichment; 10 < EF < 25 define a severe enrichment condition; 25 < EF < 50 is very severe enrichment; EF > 50 is extremely severe enrichment (Chen et al., 2007).

#### 2.7.2 Geo-accumulation Index

To determine whether the pXRF could reliably indicate metal accumulation, the Müller, (1969) geo-accumulation index was used. The $I_{\text{geo}}$ is described as the following:

(2) $$I_{\mathrm{geo}\;}={log}_{2}(\frac{C_{n}}{1.5B_{n}})$$

Where, $C_{n}$ is the mean concentration of the measured element by pXRF or ICP-MS and $B_{n}$ is the geochemical background concentration of the corresponding metal taken from Shacklette and Boerngen, (1984). The approach evaluates the metal contamination through six accumulation grades from, uncontaminated, $I_{\text{geo}}\leq 0$); very low and low contaminated $\left(0<I_{\text{geo}}\leq 1\right)$; moderately contaminated $\left(1<I_{geo}\leq 2\right)$; highly contaminated $\left(2<I_{geo}<3\right)$; very highly contaminated $\left(3<I_{geo}\right.\leq 4)$; highly to extremely contaminated $\left(4<I_{geo}\leq 5\right)$; extremely contaminated at $I_{geo}>5$ (Chen et al., 2007).

#### 2.7.2 Pollution Load Index

Pollution Load Index (PLI) provides a comparative estimate of the levels of HMs using reference values such as those provided by the U.S. Department of the Interior. The PLI helps test the impact of the HM detected by pXRF and ICP-MS on soil micro flora and fauna. To determine whether the pXRF could reliably provide a pollution load index (PLI), defined as the contamination status of each metal in relation to background concentrations at a specific site. A PLI value above 1 indicates soil pollution (Tomlinson et al., 1980). The PLI equation describes the overall risk of metal(loid)s exposures from the soil as the following:

(3) $$PLI={\left(CF_{1}\times CF_{2}\times CF_{3}\times \ldots \times CF_{n}\right)}^{\frac{1}{n}}$$

Where CF is referred as the mean ratio of the concerned metals to their background concentrations taken from United States Geological Survey (Table 2, Shacklette and Boerngen, 1984) and n is the number of total metals.

Descriptive statistics of the metal(loid)s concentrations determined by pXRF and ICP-MS in Arizona and New York soils are presented in table 3. The mean value of each element was used for EF, $I_{\text{geo}}$, and PLI calculations.

## 3. Results

### 3.1 Two-sample t-test, ICC, and R^2^

Two-sample t-test, cumulative probabilities (p-value), interclass correlation coefficients (ICC), and R^2^ results for each metal(loid)s are presented in Table 4. Arsenic, Cu, Pb, Mn, and Zn had a p>0.05, indicating a failure to reject the null hypothesis; hence, pXRF and ICP-MS do not produce significantly different measurements. Contrastingly, Ni, Ca, Cr, Fe, and K were rejected by the null hypothesis due to a p<0.05. Calcium was the only metal with a low p-value and a very strong ICC.

With regards to the Troy, NY soil samples, As, Ni, Cu, Zn, Pb, and Mn had a p>0.05. In contrast, Al, Cr, Fe, and V had a p<0.05, indicating significant mean differences. Iron was the only element with a low p-value and a very strong ICC. In addition, Al had the weakest relation between the two methods, while As, Cr, Ni, and V presented a moderate ICC, followed by Fe and Cu. The strongest correlation was exhibited by Mn, Zn, and Pb.

### 3.2 Bland-Altman and Tukey test analysis

**Figure 2.**
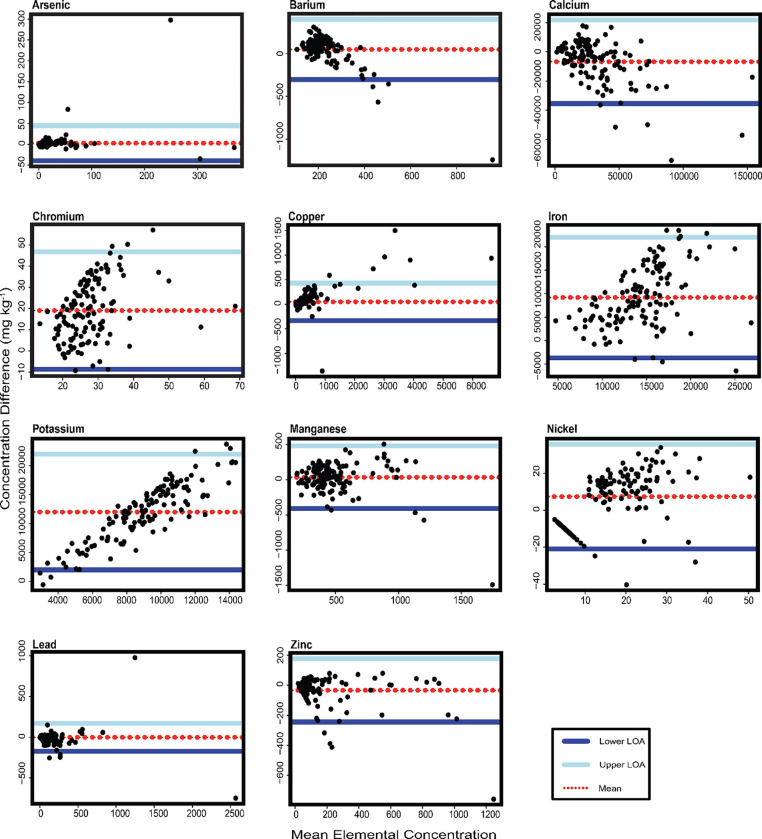
Bland-Altman plots for each element measured in Arizona soil samples. The x-axis represents the mean value of both methods, and the y-axis indicates the differences between their measurements. The upper and lower limits of agreement (LoA) indicate the range in which 95% of the values from the dataset lie. The LoA is the mean difference ± 1.96 multiply by the standard deviation of the differences.

Figure 2 shows the Bland-Altman plots for each element measured in Arizona soil samples. In general, points located around the mean line indicate no systematic biases, while points close to one of the LoA lines indicates a bias toward one method over the other. Elements with points scattered around the middle line, such as Zn, indicate no bias toward one method over the other, meaning they are similar; however, the pXRF slightly underestimates the Zn concentration. In Ca, Ba, and Cu, most points are scattered in the middle withfew points are located outside the LoA which can be due to higher concentration in one method than the other or an error in measuring. Further, the pXRF slightly overestimated the concentrations of Ba and Cu and slightly underestimated the Ca concentration. The lower and upper LoA explain the correlation strength between the two methods. A wide LoA range suggests a weak agreement as in Fe and Cr. A narrower LoA range indicates a more robust agreement as represented in Pb, Cu, As. Mn, although a few more points are in the upper LoA, explaining the slight overestimation by the pXRF. Points that form a straight line indicate a slight variation in means between the sampling methods and points scatter to form a sloped-like line, i.e., K and Ni, present a high variation between the two methods; hence, pXRF exceedingly overestimated K concentration.

**Figure. 3.**
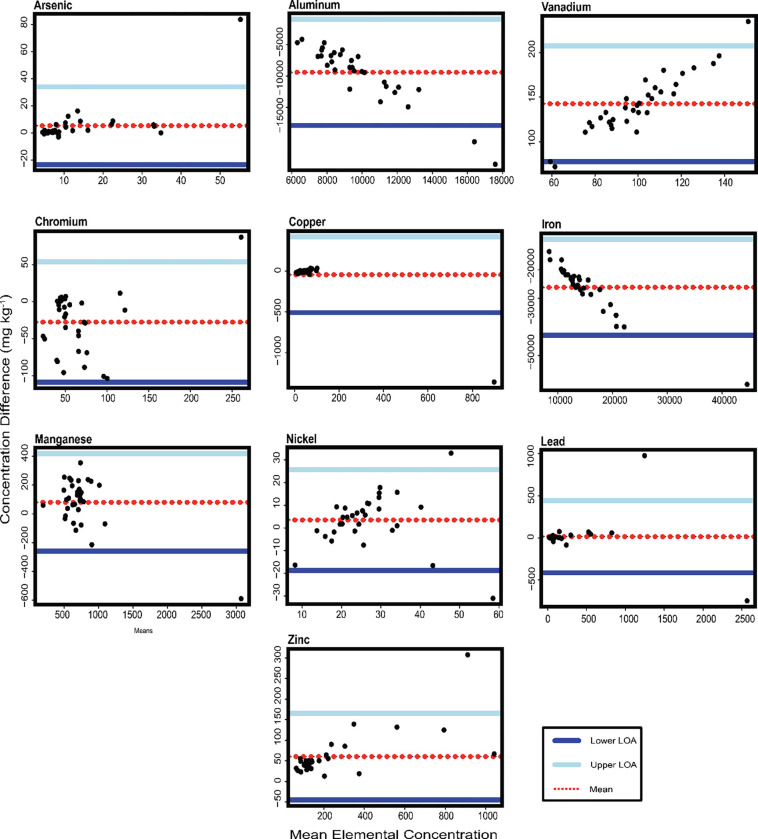
Bland-Altman plots for each element measured in New York soil samples. Bland-Altman plots for each element measured in Arizona soil samples. The x-axis represents the mean value of both methods, and the y-axis indicates the differences between their measurements. The upper and lower limits of agreement (LoA) indicate the range in which 95% of the values from the dataset lie. The LoA is the mean difference ± 1.96 multiply by the standard deviation of the differences.

Figure 3 shows the Bland-Altman plots for each element measured in New York soil samples. In New York, Pb and Cu points were scatting around the mean line, suggesting no bias toward one method over the other. Zn and As points are close to the middle and the lower LoA, representing a slight overestimation for As concentration in pXRF. The slight proportional difference in Zn means values increased the LoA between the two measurements. Accordingly, the lower concentration data are closer to each other through the pXRF measurements, which was the reason for the slight Zn overestimation. A negative slope line was formed in Fe and Al, indicating a high variation between the two methods, and possibly explained by the greater pXRF measurements when compared to ICP-MS. Further, points that formed a positive slope and scatter away from the mean line also stipulated high variation, possibly explained by overestimated pXRF concentration; e.g., Ni. Finally, Mn and Cr had the points distributed within the LoA, demonstrating a robust agreement for Mn and to a lesser extent for Cr.

When element failed at least one of the statistical analyses listed above, the Arizona samples were divided by “garden and “Yard” and the average concentration by method were compared using a post-hoc Tukey’s HSD for a two-way ANOVA. Cr, Fe, and K concentrations in yard and garden soils differed significantly, for both methods, with yard soils being greater than the garden. Ni was significantly higher in the yard for pXRF and had no different variation in the garden site. Ca and Ba concentrations were not significantly different by site type or methods.

### 3.3 Geoaccumulation Index, Enrichment Factor, and Pollution Load Index

The $I_{\text{geo}}$ and EF values are presented in table 5 for both Arizona and New York. In Arizona, the $I_{\text{geo}}$ values of Ba, As, and Mn, for both methods and locations corresponded to uncontaminated soil conditions. Cu had the highest $I_{\text{geo}}$ in yard soils followed by Pb and both fell within the range of very low and low contamination. In garden, the ICP-MS had a very low contamination index for Zn ($I_{\text{geo}}=1.24$), while presented an uncontaminated status by the pXRF ($I_{\text{geo}}=0.86$). Conversely, Zn in pXRF was analogous to ICP-MS and was showing a very low and low contamination in yard soil samples. Overall, the Arizona $I_{\text{geo}}$ values for the two methods were similar, except for Zn in garden. and the New York $I_{\text{geo}}$ values for the two methods were similar for Pb and Mn. The low As, Cu, and Zn New York $I_{\text{geo}}$ values varied by one magnitude of accumulation based on the method.

The degree of enrichment for As, Ba, Cu, Pb, and Mn were similar in both methods in Arizona, while in yard soil, Pb EF value was slightly higher in ICP-MS than pXRF. The mean EF of soil samples presented no enrichment for Ba and As, minor enrichment for Mn and Zn, and moderate enrichment for Cu in both locations. The latter had showed the highest magnitude of enrichment in Arizona. Similarly, Pb was moderately enriched in the yard samples analyzed via ICP-MS. Conversely, similar degree of enrichment for all the element in New York were observed in both methods as shown in table 5. Here, Pb came up with the highest EF value whereas As showed no enrichment through all soil samples. Mn, Cu, and Zn were mildly enriched across both methods.

Table 6 summarizes the pollution load indices for both Arizona and New York soils by of method. Pollution load indices for As, Ba, Cu, Pb, Zn and Mn in both methods were similar and exceeded average natural background concentrations. In New York, the PLI was found to be higher than Arizona for both methods; therefore, the index has provided summative indication of the overall extent of metal(loid)s pollution presented in soil.

## 4. Discussion

### 4.1 Elements with Poor Detection and Accuracy

With regards to the Arizona garden and yard samples, Co, Sb, Mo, Ag, Cd, Sn, and Sb concentrations were below the pXRF detection limits. With regards to Ni, the negative slope seems to be evident in Bland-Altman analysis, indicating a high variation between methods due to overestimation of the metal by pXRF. Additionally, Fe, K, and Cr pXRF concentrations were not correlated with the ICP-MS data. These three elemental concentrations were overestimated by pXRF with yard being noticeably higher than garden, indicating a bias towards pXRF, especially as concentrations increased. This is further supported by the low agreement between the methods (i.e., more outliers are found toward the upper LoA).

Nickel had only one point below LoA (New York) and the overall trend of the pXRF measurements for Ni and V were weakly aligned with the ICP-MS. The Ni values from Arizona and New York behaved differently and this can be linked to the higher Fe concentration. pXRF can have a spectral interferences between Fe, Co, and Ni, specifically if Fe is presented at high concentration, limiting the instrument’s ability to distinguish between the three metals (Zheng et al., 2022; Arthur & Scherer, 2020; U.S.EPA, 2007). The apparent positive slope in Bland-Altman for V has presented a bias toward pXRF. Similarly, Al and Fe had a negative trend; hence, the pXRF has underestimated the metal(loid)s levels as indicated by the higher mean concentration in the Bland-Altman plot.

Spectral interference is a common challenge when it comes to detecting lighter elements and can lower the pXRF performance (Gallhofer & Lottermoser, 2018; Declercq et al., 2019). Al is known to be a light element, making the pXRF prone to detection issues, due to the low spectrum being absorbed before reaching the pXRF detector. This is clearly observed for Al concentration above 10,000 μg g^−1^ in figure 2.

### 4.2 Elements with Moderate to Excellent accuracy

The pXRF measurements for As, Cu, Pb, Zn, Mn, and Ba had no significant differences (p >0.05) from the ICP-MS in Arizona soils. The variation in the data distribution, for example, Ba has a low R^2^, ICC, and high p-values; this phenomenon can be attributed to the decreasing trend in the Bland-Altman plot at concentrations between 320 to 510 μg g^−1^. The Tukey HSD analysis of both garden and yard data in Arizona had no significant variability of Ba, showing a better agreement between the two methods.

Based on the R^2^ interpretation the seven metal(loid)s in Arizona can be approximately ranked from highest to lowest methodological agreement: Cu>Pb>Zn>As>Ca>Mn>Ba. Although Ca had a probability of zero, the ANOVA test indicated non-significant results in both garden and yard; and there was a good agreement between the two methods through the Bland-Altman analysis.. pXRF measurements of Pb, As, and Cu were the most closely aligned with those of ICP-MS. In addition, the Bland-Altman and R^2^ of Zn and Mn had strong agreement and presented ICC of 0.91 and 0.64, respectively.

The slight overestimation of Zn in NY soil through pXRF is possibly related to the calibration mode (Yang et al., 2020) used as well as the higher mean concentration (more than 200 mg kg^−1^) as determined through Bland-Altman. That’s been said, Zn concentrations were agreeable between methods; best expressed by the R^2^ and ICC. Based on R^2^ value in New York, the quantified metal(loid)s can be ranked from the strongest to the lowest as Zn>Mn>Pb>Cu>As. In addition, As came up with the lowest R^2^, but it did not show a bias pattern for one method over the other and most detections occurred at lower mean concentrations which were close to the Bland-Altman mean line. Moreover, Pb and Cu had the best agreement demonstrated by the points scattered around the Bland-Altman mean line, revealing a very strong pXRF accuracy.

### 4.3 Challenges associated with select soil properties and the pXRF

**Figure 4.**
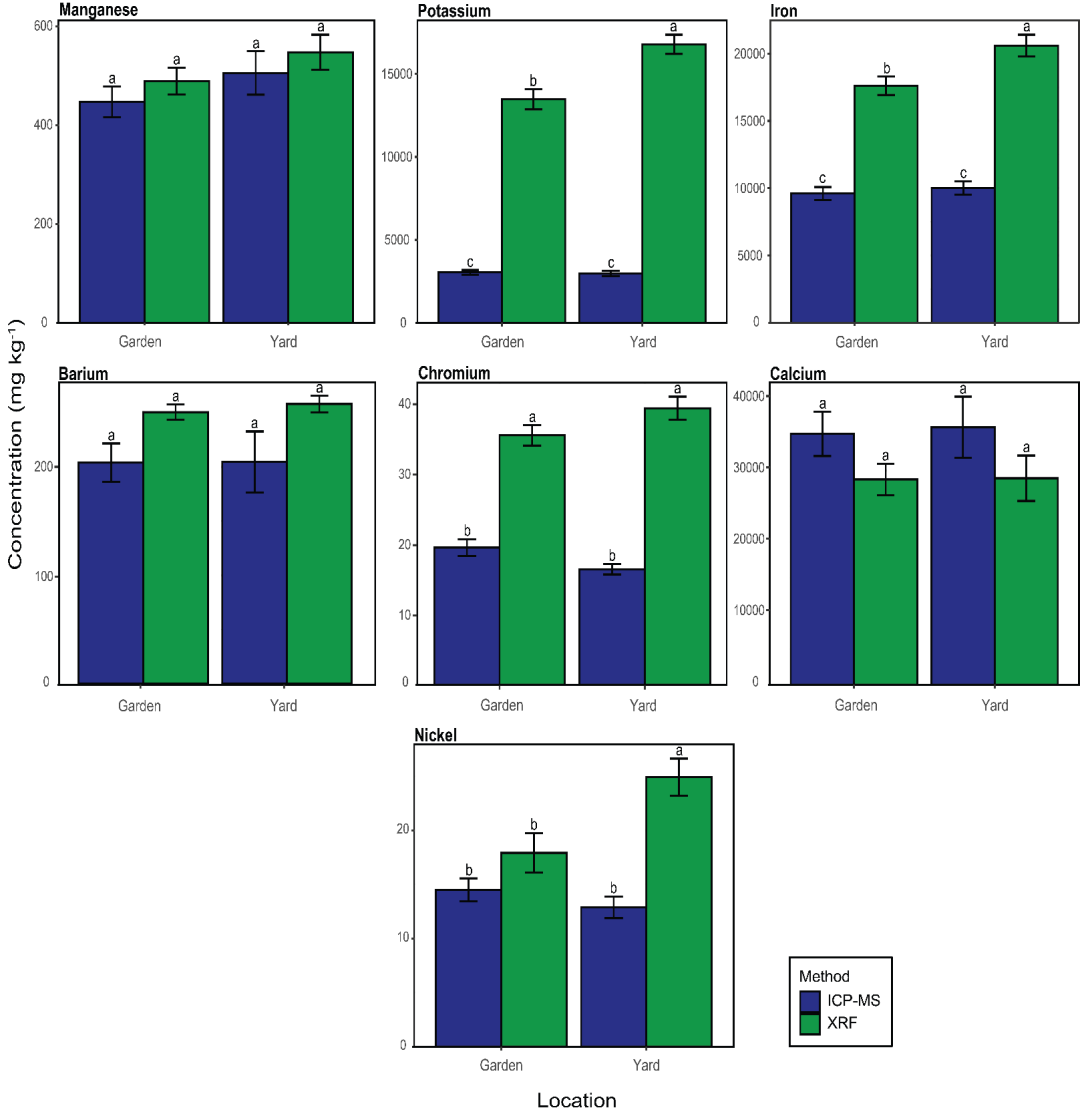
Bar plots and of mean elemental Arizona garden and yard soil concentrations in by method. The error line in the figure represents the standard deviation. A Tukey’s HSD post-hoc test for a two-factor ANOVA was used; bars with the different letters indicate a significant difference.

With regards to the comparison of yard vs. garden, the application of soil amendments can increase the amount of organic matter and constant irrigation can readily leach available elements throughout the soil horizons. Figure 4 shows the discrepancy between the two methods, where the gardens’ metal(loid)s concentrations are less than yard. Although the result was different between the two sites, here can possibly relate the lower Cr measured by pXRF in garden to soil OM; Ravansari, (2016) observed that the Cr concentration measured by pXRF decreased with the increase in cellulose organic matter fraction (Ravansari, 2016). Additionally, Ravansari and Lemke, (2018) had discussed the concentration deviations presented by pXRF based on the addition of different OM fractions. The attenuation response was elementally dependent on the increase of OM fractions. This scenario was attributed to the mode of calibration and pXRF algorithms and both were built upon the soil metrics provided by the manufactory.

**Figure. 5.**
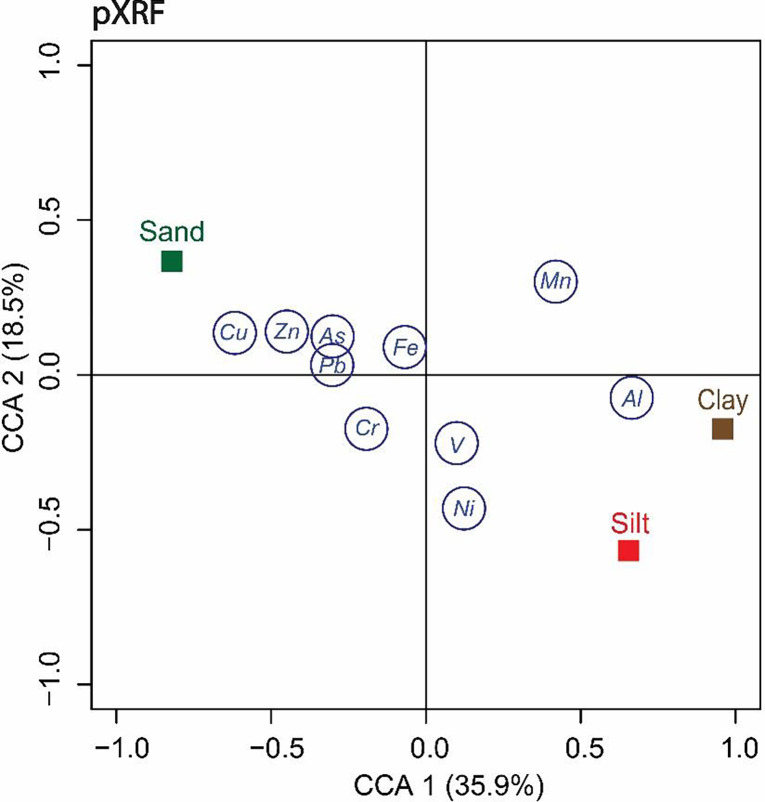
Canonical Correspondence Analysis Diagram showing the association between soil texture and pXRF elemental concentrations in soil from Troy, New York. Chromium and Manganese were not located in proximity to any soil texture, indicating an unclear association between the pXRF measurement and soil texture.

The CCA diagram revealed the correlation between soil texture analysis and pXRF elemental measurements in Troy, New York (Fig. 5). The first two principal dimensions CCA1 and CCA2 explained 35.9% and 18.5 % of the total variance, respectively. A positive correlation was observed between sand and Cu, Zn, As, Pb, and Fe; clay and Al, V, and Ni; Silt and Al, Ni, and V. Here, one is expecting Cr to be positively correlated to soil texture (Kim & Dixon, 2002; Lacroix et al., 2021; Rudzionis et al., 2022), however, due to high Fe concentration, the pXRF efficiency in reporting the actual amount of Cr declines due to a lower absorption edge in energy than the fluorescent peak of Fe (EPA, 2007). Such an effect can be corrected mathematically using fundamental parameter coefficients related to particle size and matrix effects. The consequences of calibrating pXRF by LOD has been widely studied, recent work has shown the disparities in X-ray spectrum for non-quantified elements, necessitating the manual inspection and calibration of the pXRF (Singh et al., 2022). On that account, the attenuation in pXRF measurements caused by OM needs to be further investigated to validate the technique’s calibration, namely in amended soil. Arsenic and Pb have a dependent relationship, high lead soil concentrations will affect the pXRF spectra detection range of As, which is described by the manufacturer as Interference-free detection limits (DLs) (Olympus Corporation, n.d.b.). Here, the L-lines emitted by atoms of Pb overlap with the K-line of As (Gallhofer and Lottermoser, 2018). The pXRF model attempts to automatically correct the As value when Pb is presented in high concentrations; however, in these instances, is critical to manually calibrate the instrument with soil from the targeted region to improve As detection.

### 4.4 The influence of anthropogenic activities

As described in section 2.1, mining activities may have affected some areas of Arizona and releasing heavy metals in and surrounding communities. Some As compounds and ions are distributed in the surrounding environment during smelting and mining the ore, impacting nearby communities, primarily via surface soil deposition, impacting residential areas (Sutherland et al., 2003). Zinc enrichment was observed, indicating anthropogenic activities influencing soil concentrations. This is especially observed in the enrichment analyses conducted with the pXRF garden’s data. The result might be related to the mining industry in Greenlee County, Arizona. Using the U.S.EPA Toxic Release Inventory (U.S.EPA TRI) data set, the risk-screening environmental indicator reported a median released or transferred of 7,199 pounds for Cu, Ni, Pb, and Zn together, this is 24 times higher than the reported state median value (U.S.EPA, 2021). Lead had a different $I_{\text{geo}}$ description in Arizona yard than garden in pXRF, and yard in ICP-MS. The discrepancy within the pXRF might be related to different sources of Pb. Troy is an urban area influenced by anthropogenic activity, i.e., roadside soil accumulates Pb due to car exhaust emissions and in general, soils are impacted by the atmospheric deposition of Pb, Pb-based paint, and ongoing industrial activities (Ravansari et al., 2020; Turner and Lewis, 2018; Wang et al. 2006). Arsenic measured by pXRF had a higher magnitude of $I_{\text{geo}}$ than ICP-MS in Troy, NY, which may be attributed to the difference in anthropogenic sources since samples were collected across the city. With regards to the PLI it is important to note that the metal(loid)s measured in this study may be naturally occurring due to local geologic conditions where formed soils may have naturally elevated levels of certain metal(loid)s.

## 5. Conclusion

The elevated accumulation rate of metal(loid)s in soils presents a potential risk to human health, especially when little attention is given to soil health as related to local geology and the potential impact of anthropogenic activities. This calls for raising community awareness and increasing capacity to take appropriate environmental monitoring measures. This effort requires a method like the pXRF that is viable for use, relatively low cost, and user-friendly.

The assessment of 19 elements divided between Arizona and New York highlighted the pXRF reliability to measure As, Pb, Cu, Zn, Ca, Ba and Mn. The dynamic statistical approach employed in this study demonstrated a correlation between pXRF and ICP-MS measurements. The discrepancies in the agreement between the two methods can be minimized by properly calibrating the instrument based on the area of interest’s soil matrix. Moreover, the evidence and observation from other studies had previously reported the pXRF failures based on spectra interference between non-quantified metal(loid)s, like Ni and Fe. Similarly, pXRF had failed to detect Al and presented a significant variance compared to ICP-MS due to its light atomic weight.

The proposed study is building upon the Gardenroot project methodology (Ramírez-Andreotta et al., 2015), which works alongside local communities near resource extraction sites to build human capacity, increase our understanding of their surrounding environment, and provide public health intervention and prevention practices to mitigate/minimize metal(loid) exposures and risk. Here, the data was governed by the resources available such as community participation. Since efforts focused on exposure science public health prevention/intervention strategies, other variables like pH, OM, and PSD were not determined. Future efforts should include more soil biogeochemical analyses and pre-calibration techniques to further tease out the disparities between pXRF and the gold standard, ICP-MS to extend the application of pXRF device. Regardless, this study highlights the pXRF reliability to measure As, Ba, Ca, Cu, Mn, Pb, and Zn indicating its utility in community soil monitoring efforts.

## Figures and Tables

**Table 1.** Limits of detection for pXRF (DELTA Premium, DP-6000) and ICP-MS (ppm/µg g^−1^). The ICP-MS methodological limit of detection (MDL) for each element was calculated based on the instrument detection limit after applying the dilution factor.

pXRF *											
Element	As	Ni	Ca	Cu	Cr	Ba	Fe	K	Pb	Mn	Zn
**LOD**	1–3	4–10	10–35	2–6	2–9	15–30	5–20	20–50	1–4	3–7	1–3
ICP-MS											
Element	As	Ni	Ca	Cu	Cr	Ba	Fe	K	Pb	Mn	Zn
**MDL**	0.027	0.067	1.140	0.030	0.021	0.002	0.034	4.206	0.004	0.006	0.023

* Limit of detections for soils and the geochemical modes (Olympus Corporation, n.d.c).

**Table 2.** Background elemental soil concentrations (µg g^−1^) for the western conterminous states, USA as originally provided by Shacklette & Boerngen, 1984.

Elements				
	Minimum	Maximum	Mean	SD

As	0.1	97	5.5	1.98

Ba	70	5000	580	1.72

Cu	2	300	21	2.07

Pb	10	700	17	1.8

Mn	30	5000	380	1.98

Zn	10	2100	55	1.79

**Table 3.** Elemental Arizona and New York soil concentrations (µg g^−1^) determined by pXRF and ICP-MS analysis.

Arizona								

	ICP-MS (N=124)				pXRF (N=124)			
Elements	Minimum	Maximum	Mean	SD	Minimum	Maximum	Mean	SD

Zn	13.8	1626	187.1	252.1	16.8	908.7	152.7	213.2
Mn	149.6	2493.3	478.3	287	118.7	1264	511.5	240.9
Pb	4.65	498.9	56.8	93.4	5.63	436	51.4	71.5
As	0.79	23.2	4.6	3.27	2.15	26.7	6.27	4.1
Ba*	24.4	1576	204.9	178.4	129.7	417	253.6	55.6
Ca	1406	1.7×10^5^	3.5×10^5^	2.9×10^4^	1277	1.5×10^5^	2.9×10^4^	2.1×10^4^
Cu	5.78	1019	105.8	174.7	6.8	1129	116.7	186
Ni	2.17	51	13.8	8.2	15	59.3	28.2	8.94
Cr	6.46	58.2	18.05	8.27	19	79.3	37.2	12.2
K	772.9	6820	3008	1186	2836	2.6×10^4^	1.5×10^4^	4980
Fe	2614	2.8×10^4^	9793	3865	6838	3.5×10^4^	1.9×10^4^	6012
								

New York								

	ICP-MS (N=33)				pXRF (N=33)			
	
Elements	Minimum	Maximum	Mean	SD	Minimum	Maximum	Mean	SD
Zn	3.9	1008	198.3	224.6	79.7	1075	264.8	262.7
Mn	47.8	3362	689.7	513.7	223.5	2773	789.5	395.7
Pb	2.1	2941	227.5	520.1	21	2194	249.5	482.9
As	0.9	34.7	9.8	7.96	4.58	97	15.5	17.3
Al	2439	3 ×10^4^	1.4×10^4^	4974	3251	7148	5113	840.1
V	5.9	43.9	28.1	8.2	97.4	268.4	171.4	36.4
Cu	4.1	1577	86.5	264.3	11.9	218	51.5	44.4
Ni	5.	74	24.1	11.8	13.1	64.3	29.1	10.6
Cr	40.1	217	78.5	39.7	28.5	304.1	59.9	52.2
Fe	3806	7.5×10^4^	2.7×10^4^	1.1×10^4^	2.3×10^4^	7.2×10^4^	3.3×10^4^	9487

* Barium was only measured in Arizona due to limited number of New York soil samples.

**Table 4.** Two-sample t-test, interclass correlation coefficients (ICC), and R^2^ results for each element of interest. Values were obtained using a 95% confidence level and a two-way agreement model. Bolded text indicates statistical significance.

	Arizona										
	
	As	Ni	Ca	Cu	Cr	Ba	Fe	Zn	Pb	Mn	K

ICC coefficients	0.87	0.29	0.87	0.98	0.09	0.13	0.17	0.91	0.92	0.64	0.02
P-value	0.45	**0**	**0.04**	0.42	**0**	0.17	**0**	0.24	0.97	0.33	**0**
T- Statistic	0.74	−4.93	2.11	0.81	−14.43	1.39	−14.32	1.16	0.04	0.99	26.08
R^2^	0.76	0.08	0.76	0.95	0.01	0.02	0.03	0.82	0.83	0.42	0

	New York									
	
	As	Ni	V	Cu	Cr	Al	Fe	Zn	Pb	Mn	

ICC coefficients	0.54	0.52	0.53	0.73	0.63	0.40	0.81	0.99	0.91	0.95	
P-value	0.11	0.22	**0**	0.38	**0.02**	**0**	**0**	0.31	0.90	0.47	
T- Statistic	1.62	1.24	22.09	−0.89	−2.39	−11.6	−13.9	1	0.12	0.72	
R^2^	0.29	0.27	0.28	0.54	0.40	0.16	0.66	0.98	0.83	0.92	

**Table 5.** Arizona and Troy, New York, USA soil geoaccumulation indices ($I_{\text{geo}}$) and enrichment factors (EF) by pXRF and ICP-MS method (mean metal(loid) concentrations were used in calculations). A color gradient is used to indicate contamination (orange) and enrichment (blue).

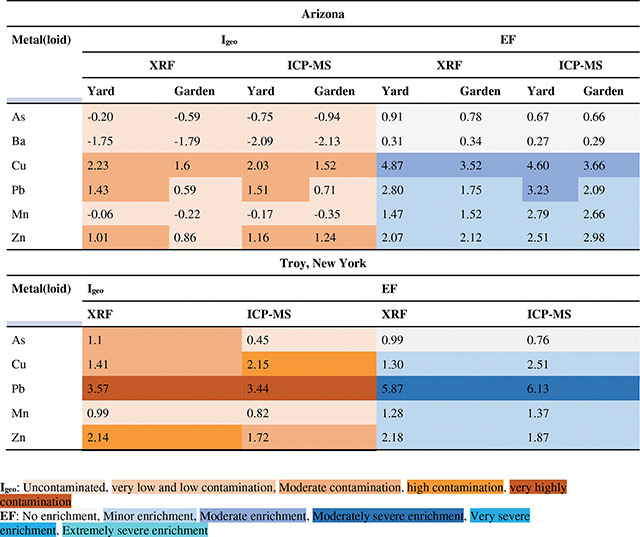

**Table 6.** Arizona and Troy, New York, USA soil pollution load indices (PLI) by pXRF and ICP-MS method (mean metal(loid) concentrations were used in calculations). A similar PLI value indicates the reliability of pXRF to closely describe the pollution status.

	Arizona			

PLI	XRF		ICP-MS	
	
	Yard	Garden	Yard	Garden

**Value**	1.59	1.53	1.61	1.52
**Contamination Status** *	Polluted	Polluted	Polluted	Polluted

	Troy, New York			

PLI	XRF		ICP-MS	

**Value**	2.01		2.03	
**Contamination Status**	Polluted		Polluted	

* The pollution index calculation combines all elements and due to this summation, some elements can be responsible for driving the pollution index, such as Zn and Cu. This is apparent in the calculated $I_{\text{geo}}$ values where Zn and Cu have a moderate degree of accumulation in Arizona soil. Similarly, Cu is moderately enriched in Arizona soil as indicated in Table 5.

## Data Availability

Datasets can be requested from the corresponding author.
